# O.1.3-5 A review of NHS Scotland policies and strategies on employee wellbeing: the promotion of physical activity

**DOI:** 10.1093/eurpub/ckad133.102

**Published:** 2023-09-11

**Authors:** Gemma Cathrine Ryde, Louise Patricia Hoyle

**Affiliations:** University of Glasgow, UK; University of Stirling, UK; gemma.ryde@glasgow.ac.uk

## Abstract

**Purpose:**

The NHS in Scotland is under increasing strain to deliver essential health services since the 2019 COVID. This is in part due to pressures caused by employee absences which even before the pandemic were increasing and at a higher rate than the national average. Promoting lifestyle choices such as physical activity to NHS employees can potentially improve this issue. However, the extent to which employees are encourage and supported to be active at a policy or strategic level is unknown. The aim of this study was to assess the number of health boards that had a policy or strategy document focusing on employee health and to assess the extent to which physical activity was part of this.

**Methods:**

Freedom of Information Requests (FOI) were sent to all 14 Scottish NHS Health Boards to request any workplace documents (both strategic and non-strategic) which focus on employee health and wellbeing. Online searches were also conducted for each health board to look for any additional documents related to staff wellbeing. After removing duplicates, titles and content were assessed for relevance to employee health and wellbeing. The content of include documents were reviewed and any mention of physical activity recorded.

**Results:**

Thirteen documents were retrieved with 11 of the 14 Health Boards having a at least one policy or strategy document that focused on employee health and wellbeing. The oldest document dated back to 2013 with four published since 2020. Of the 13 documents, nine documents mention physical activity but mainly in relation to current activities rather than in the context of a future healthy workforce.

**Conclusion:**

Despite the importance of a healthy NHS workforce, some Health Boards still do not have policy or strategy documents to support this. Those that do are potentially limited by the lack of reference to lifestyle behaviours such as physical activity. In order to improve employee health and wellbeing in the NHS, organisational level documents that encourage health behaviours for staff such as increasing physical activity are needed.

